# Epidemiologic, Postmortem Computed Tomography-Morphologic and Biomechanical Analysis of the Effects of Non-Invasive External Pelvic Stabilizers in Genuine Unstable Pelvic Injuries

**DOI:** 10.3390/jcm10194348

**Published:** 2021-09-24

**Authors:** Christian Kleber, Mirja Haussmann, Michael Hetz, Michael Tsokos, Claas T. Buschmann

**Affiliations:** 1 University Center of Orthopaedic, Trauma and Plastic Surgery, University Hospital Carl Gustav Carus, 01307 Dresden, Germany; Michael.Hetz@uniklinikum-dresden.de; 2 Department of Anesthesiology and Operative Intensive Care Medicine, Charité-Universiätsmedizin, 10117 Berlin, Germany; mirja.haussmann@charite.de; 3 Institute of Legal Medicine and Forensic Sciences, Charité-Universiätsmedizin, 10117 Berlin, Germany; michael.tsokos@charite.de (M.T.); Claas.Buschmann@uksh.de (C.T.B.); 4 Institute of Legal Medicine and Forensic Sciences, UKSH Universitätsklinikum Schleswig-Holstein, 24105 Kiel, Germany

**Keywords:** trauma, non-invasive external pelvic stabilizers, bleeding, pelvic fractures, post mortem analysis, biomechanical force, pneumatic pelvic sling VBM^®^, T-POD^®^, cloth sling, SAM Sling^®^

## Abstract

Unstable pelvic injuries are rare (3–8% of all fractures) but are associated with a mortality of up to 30%. An effective way to treat venous and cancellous sources of bleeding prehospital is to reduce intrapelvic volume with external noninvasive pelvic stabilizers. Scientifically reliable data regarding pelvic volume reduction and applicable pressure are lacking. Epidemiologic data were collected, and multiple post-mortem CT scans and biomechanical measurements were performed on real, unstable pelvic injuries. Unstable pelvic injury was shown to be the leading source of bleeding in only 19%. All external non-invasive pelvic stabilizers achieved intrapelvic volume reduction; the T-POD^®^ succeeded best on average (333 ± 234 cm^3^), but with higher average peak traction (110 N). The reduction results of the VBM^®^ pneumatic pelvic sling consistently showed significantly better results at a pressure of 200 mmHg than at 100 mmHg at similar peak traction forces. All pelvic stabilizers exhibited the highest peak tensile force shortly after application. Unstable pelvic injuries must be considered as an indicator of serious concomitant injuries. Stabilization should be performed prehospital with specific pelvic stabilizers, such as the T-POD^®^ or the VBM^®^ pneumatic pelvic sling. We recommend adjusting the pressure recommendation of the VBM^®^ pneumatic pelvic sling to 200 mmHg.

## 1. Introduction

With a proportion of 2–8%, pelvic fractures represent a rare injury. They occur most frequently in the 2nd and 3rd life decades [1] and are often the result of high-energy trauma, thus appearing in up to 20% of polytrauma patients [1,2]. Complex pelvic ring fractures are associated with a mortality rate of 5–42% [3,4,5]. In many cases, the high energy trauma causes extra and intrapelvic concomitant injuries, which can be life threatening [1,6]. Despite achieving improved survival rates in recent years, the mortality of open pelvic fractures is reported at up to 70% [1,7,8,9]. The immediate risk to life is linked to the possible occurrence of refractory hemorrhagic shock, with associated major coagulation disorders [3,8,10].

The pelvic ring is anatomically connected to many blood vessels [10]. Three main sources of hemorrhage are described. These include arterial bleeding from the great arterial pelvic vessels, the venous vascular system, and exposed cancellous fracture surfaces of the posterior pelvic ring [8,11,12,13]. Auto tamponade is unlikely due to torn retroperitoneal structures with potentially massive intrapelvic or retroperitoneal blood loss, which may lead to exsanguination [1,8]. It is important for modern priority-guided trauma management to detect the leading injury and source of immediate life threat (“treat first what kills first”). Therefore, in the first part of this study we analyzed the epidemiology of genuine pelvic injuries referring to the cause of death and primary bleeding source. The application of external non-invasive external pelvic stabilization is recommended in several guidelines and is nearly the only measure to treat unstable pelvic injuries in a prehospital setting [11,14,15].

A theoretical way to minimize bleeding, especially venous and spongy sources, is to reduce the intrapelvic volume with the approximate reduction of the fracture ends and closure of the anterior/posterior pelvic ring using external noninvasive pelvic stabilizers [13,16]. This hypothesis is based on clinical experience and two studies. Tan et al. revealed improved blood pressure in a small case series after the application of external non-invasive pelvic stabilization [17]. Grimm et al. showed increased retroperitoneal pressure in an artificial cadaver pelvic injury model via closed reduction with an external fixation of the pelvic ring and an infusion of the retroperitoneum [18]. Furthermore, several studies reveal the biomechanical impact of external non-invasive pelvic stabilization in artificial models of pelvic injuries [4,19,20,21]; however, the proof to reduced intrapelvine volume in unstable pelvic injuries is still missing.

Therefore, in the second part of this study, we analyzed for the first time the effects of different external noninvasive pelvic stabilizers on intrapelvic volume in real pelvic injuries using a postmortem CT scan and compared their effects on pelvic biomechanics.

## 2. Materials and Methods

This study is divided into a retrospective case series to collect epidemiologic data and a prospective intervention study to analyze the effect and biomechanical properties of external noninvasive pelvic stabilizers (Ethics Vote EA1/250/11). Substantial elements of this script are based on the dissertation by Dr. Haussmann [22].

In the retrospective part, all cases of deceased patients with mechanically unstable pelvic injuries in the archives of the Institute of Legal Medicine and Forensic Sciences, Charité-Universiätsmedizin, Berlin, were analyzed (n = 91). The survey period was 3 January 2012 to 30 September 2013. In addition to age and sex, accident mechanism, preclinical measures, and the place of death were investigated. Furthermore, the autopsy protocols were used to ascertain the cause of death, the leading bleeding source, and vascular injury in the abdominal and pelvic regions. Peripelvic bleeding was defined as bleeding sources around the bony pelvis, including vessel branches of the internal/external iliac vessels, muscles, soft tissue, and skin.

The second prospective interventional part of the study with the application of external pelvic stabilization and CT-guided measurements were performed from 3 January 2012 to 30 September 2013 (n = 36).

The inclusion criteria for both subprojects were legally authorized autopsy, death after traumatic injury, pelvic instability in physical examination, minimum age of 18 years, preserved integrity of the peripelvic soft tissues, and the absence of osteosynthetic treatment. Exclusion criteria were the emergency operations and invasive pelvic stabilization. The external pelvic stabilization devices tested were the following:

− The pneumatic pelvic sling Standard^®^ (100 and 200 mmHg; VBM Medizintechnik GmbH, Sulz, Germany)
− T-POD^®^ (Pyng Medical, Richmond, ON, Canada)
− Conventional cloth sling (bed sheet)
− SAM Pelvic Sling II^®^ (SAM Medical Products, Wilsonville, OR, USA)

All devices were applied according to the study of Bottlang et al. at the level of the greater trochanters [4].

The breaking of rigor mortis was followed by the placement of the three devices for provisional external pelvic stabilization for the respective computed tomographic documentation of the compression effect of the fractured pelvis. The cranial limit of the scan area was chosen for these scans at the level of the third lumbar vertebrae and caudally at the level of the middle of the femur. A native image of the selected scan area was taken immediately prior to application of the corresponding device in each case to ensure a direct before and after comparison. All CT scans of the pelvic region were taken with a slice thickness of 0.5 mm (Activion 16; Toshiba).

**Figure 1:**
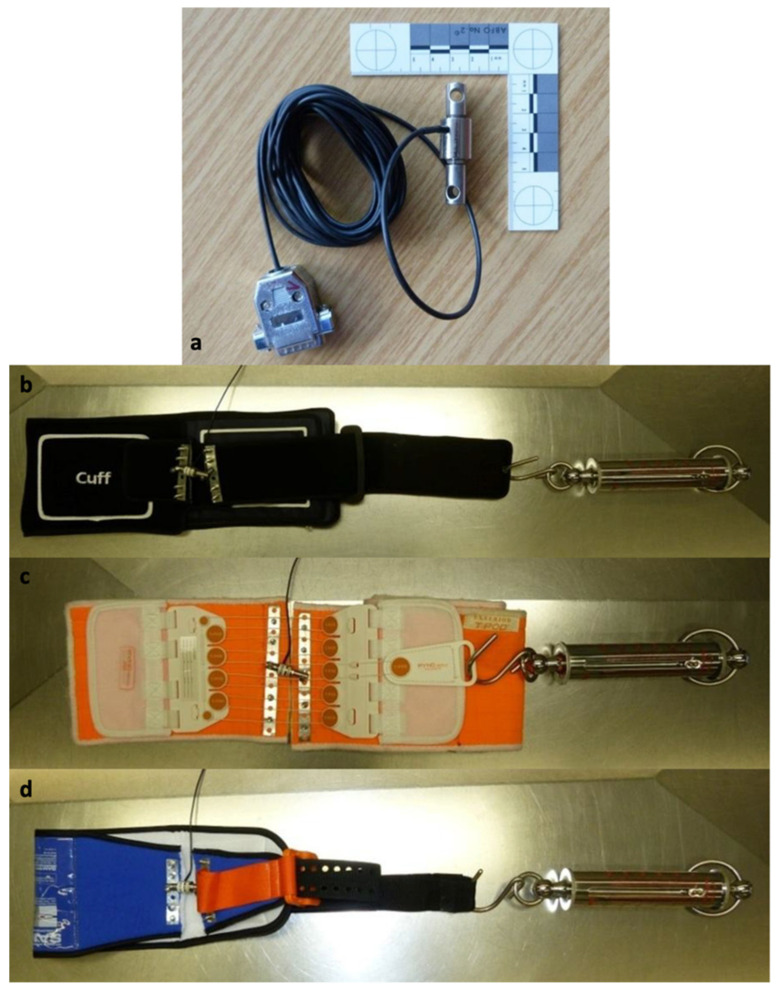
Used devices: (**a**) Newtonmeter; (**b**) Pneumatic pelvic sling VBM^®^ prepared with Newtonmeter and tension spring; (**c**) T-POD^®^ prepared with Newtonmeter and tension spring; (**d**) SAM Sling^®^ prepared with Newtonmeter and tension spring (source: Haussmann [22], modified).

To measure the traction forces, the different devices were prepared and a tension spring (Kraftaufnehmer OCDZ 0-3000N; Wazau Mess- und Prüfsysteme GmbH) was integrated (Figure 1).

The calibration of the equipment was performed by a biomechanist of the Julius-Wolff-Institute of the Charité—Universitätsmedizin.

The documentation of the applied traction forces with the pelvic sling in place during the performance of CT scans was performed at four defined time points: 45 s (t1), 80 s (t2), and 120 s (t3). The maximum achieved traction force (Fmax)—independent of the time point—was also recorded.

Before and after application of the respective pelvic stabilization device, the volumetry of the pelvic ring, area of the pelvic entrance plane, distances between the centers of both femoral heads, the Köhler’s tear figures, the sacroiliac joints ventrally and dorsally, and the distance/width of the symphysis were measured. The program OsiriX^®^ (vers. 4.1, Pixmeo, Bernex, Switzerland) was used for image analysis.

**Figure 2:**
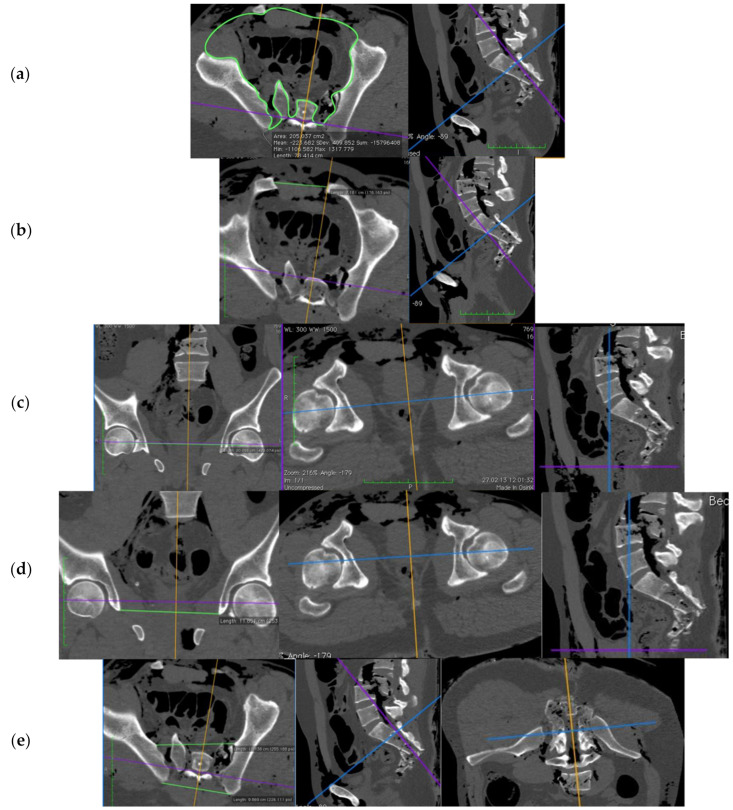
Representation of the different measurement planes and distances using OsiriX^®^: (**a**) pelvic entrance plane; (**b**) symphyseal width; (**c**) femoral head distance; (**d**) distance of Köhler’s tear figures; (**e**) ventral and dorsal distance between sacroiliac joints (source: Haussmann [22], modified).

For the volumetrics, the pelvis was primarily standardized in all three planes and distances were defined as follows (Figure 2):

− cranial to caudal: the junction between lumbar vertebrae 4 and 5 to the caudal end of the ischiatic tuber
− the area of pelvic entrance plane: the transverse plane between the lower edge of sacral vertebra 1 and the upper edge of the symphysis
− the symphysis width: the point of greatest distance between the pubic bones
− the femoral head distance: the distance between the centers of the femoral heads (in frontal plane)
− the distance between the Köhler’s tear figures: the shortest distance between the most caudal poles (in frontal plane)
− the distance between the sacroiliac joints (SIJ): the ventral portions of the SIJ space, and for the dorsal distance, the most dorsal bony border of the Os ileum.

Data processing was performed using IBM SPSS Statistics 22^®^ (IBM Corporation Armonk, NY, USA) and Microsoft Office Excel 2007^®^ (Microsoft Corporation, Redmond, WA, USA). The Wilcoxon and Kolmogorov tests were applied for non-normally distributed variables and a paired t-test for normally distributed variables. The significance was assumed at a *p* < 0.05.

CT scans were again taken in the identical, standardized sequence on a total of six patients with mechanically unstable pelvic findings in the external cadaveric examination. In addition, the measurements were now supplemented by the application of the SAM Sling^®^.

All four pelvic slings were prepared with tension springs for the CT scans to document the acting tension forces after proper application.

## 3. Results

### 3.1. Epidemiological Data

Epidemiologic data was analyzed for 91 casualties, 36 from the prospective interventional study collective, and 55 patients after file review in the archives of the Institute of Legal and Forensic Medicine, Charité-Universitätsmedizin, Berlin.

**Figure 3:**
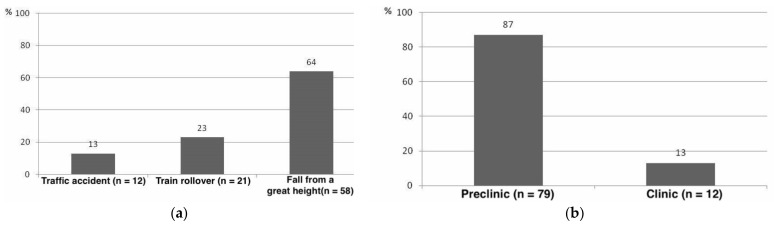
Distribution of accident mechanisms and localization of death (n = 91): (**a**) Most patients died as a result of a fall from a great height, followed by train rollovers and traffic accidents. (**b**) Majority of patients died in the prehospital setting (source: Haussmann [22], modified).

The mean age of the 91 patients was 49 ± 19 years (range 18 to 92). 67 of the patients were male. 13% (n = 12) of deaths were caused by a traffic accident, in 23% (n = 21) by train rollover trauma, and in 64% (n = 58) by a fall from a substantial height. 79 patients (87%) died prehospital, and 12 patients (13%) died in the hospital (Figure 3).

**Figure 4:**
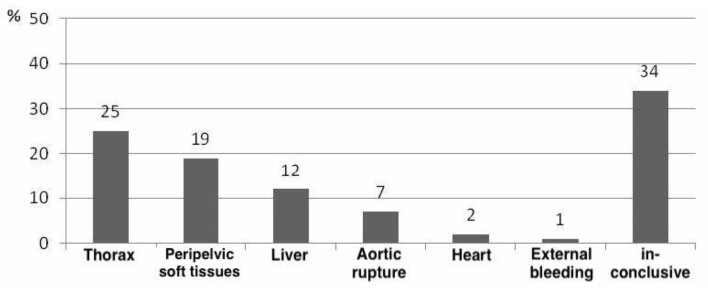
Overview of the main sources of hemorrhage in the examined collective: thoracic (25%, n = 23), followed by peripelvic (19%, n = 17) bleeding, hemorrhage of the liver (12%, n = 11), aortic rupture (7%, n = 6), and destruction of the heart (2%, n = 2). External sources of bleeding—due to transfemoral amputation—occurred in only one case (1%, n = 1). In 34% (n = 31) of the patients, a clear assignment of the main source of bleeding was not possible during autopsy due to multiple injuries. (Source: Haussmann [22], modified).

The leading sources of bleeding were thoracic, followed by peripelvic bleeding, hemorrhage of the liver, aortic rupture, and destruction of the heart. External sources of bleeding—due to transfemoral amputation—occurred in only one case. In 34% of the patients, a clear assignment of the main source of bleeding was not possible during autopsy due to multiple injuries (Figure 4).

**Figure 5:**
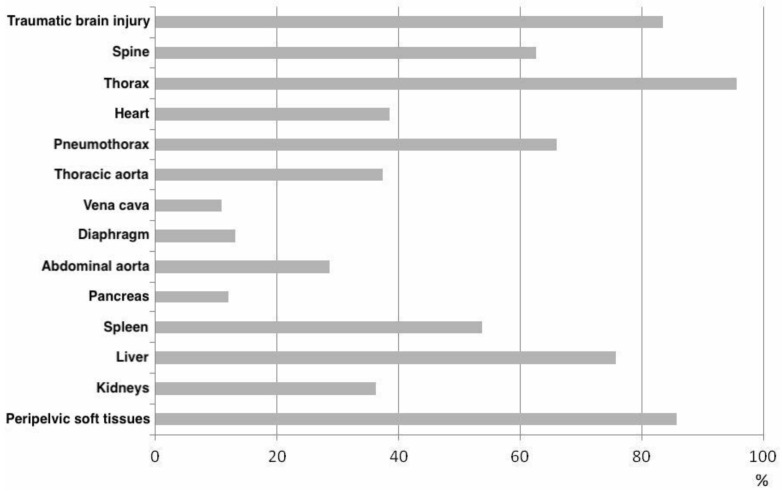
Summary of injury distribution in deceased patients with unstable pelvic injury: almost all patients showed thoracic injuries (96%, n = 87): 66% of patients (n = 60) had pneumothorax, 39% (n = 35) cardiac injury. Traumatic brain injury was documented in 84% (n = 76). Injuries to the spine in 63% (n = 57). 76% of patients (n = 69) showed injury to the liver, 54% (n = 49) to the spleen. Renal and pancreatic injuries were less frequent with 36% (n = 33) and 12% (n = 11), respectively. Diaphragmatic ruptures as a sign of extensive two-cavity trauma were detected in 13% of the collective (n = 12). In 37% of patients (n = 34), an injury to the aorta was found, in 29% (n = 26) of the thoracic aorta. In contrast, the superior or inferior vena cava was injured in 11% (n = 10). Peripelvic soft tissue damage was evident in 86% of the studied collective (n = 78). In 11% of the population (n = 10), the condition of the peripelvic soft tissues was not documented in the autopsy protocols (source: Haussmann [22], modified).

As shown in Figure 4, thoracic concomitant injuries led the way, followed by peripelvic injuries and traumatic brain injury (Figure 5).

The cause of death was mainly multiple trauma (93%, n = 85). In 6% of the cases (n = 5), death occurred by exsanguination. In one patient (1%), traumatic brain injury was the cause of death.

**Figure 6:**
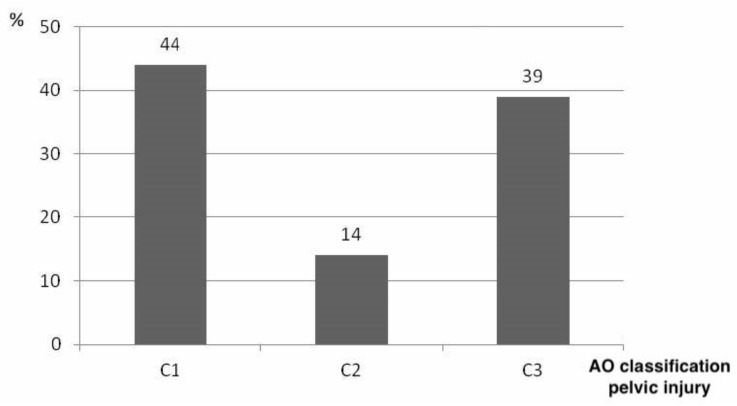
Distribution of type C pelvic injuries of the studied collective. Leading were type-C1 injuries, followed by type-C3 pelvic injuries (source: Haussmann [22], modified).

Based on our postmortem CT scans, pelvic ring injuries were classified according to AO. Type C pelvic injuries were the most common in the studied population, and their distribution is shown in Figure 6.

Type B pelvic injuries were not detected in the studied collective. One patient with a type A pelvic injury was included in the study because of the clinical impression of the unstable pelvis during clinical stability testing for Ala fracture. A postmortem CT analysis revealed that it was a type A injury.

### 3.2. Effects on Pelvic Bioarchitecture of External, Non-Invasive Pelvic Stabilizers

The following results refer to the CT scans of the study population. Table 1 serves as a summary of the average intrapelvic volume reduction results of the external noninvasive pelvic stabilizers tested:

All applied external non-invasive pelvic stabilizers were able to achieve a reduction of the area in the pelvic entrance plane, symphysis width, reduction of the femoral head distance, and reduction of the distance of the Köhler’s tear figure, reduction of the ventral and dorsal distance of the ileosacral joint.

With regard to the reduction of intrapelvic volume (PV), the pneumatic pelvic sling VBM^®^ with an applied pressure of 200 mmHg showed the best results (for detailed data please see Table 1 and Table 2). This was followed in descending order by the VBM^®^ at 100 mmHg the T-POD^®^ and the cloth sling.

The results of the area reduction of the pelvic entrance level (PA) are, in descending order: pneumatic VBM^®^ pelvic sling with 200 mmHg pressure, followed by the same with 100 mmHg, T-POD^®^ and the cloth sling.

The comparison of the cloth sling with the pneumatic VBM^®^ pelvic sling at an applied pressure of 100 mmHg showed significantly lower reduction results, regarding the reduction of symphysis width (SW).

Regarding femoral head distance (FH), the VBM^®^ pneumatic pelvic sling at 200 mmHg also achieved the best reduction results. This was followed by VBM^®^ at 100 mmHg, T-POD^®^ (*p* = 0.001), and the cloth sling.

The reduction of the distance between Köhler’s tear figures (KT) was again significantly better achieved by the pneumatic pelvic sling VBM^®^ with 200 mmHg than with an applied pressure of 100 mmHg. The T-POD^®^ followed. The cloth sling achieved significantly lower results.

Reduction of ventral ileosacral joint distance (SIJv) with the VBM^®^ pneumatic pelvic sling at 200 mmHg also produced the best results, followed by 100 mmHg, T-POD^®^, and the cloth sling.

A comparison of the reduction in dorsal ileosacral joint (SIJd) distance among the pelvic stabilizers showed no significant differences.

### 3.3. Biomechanical Force Measurement of the Acting Tensile Forces after Pelvic Stabilizer Device

The average peak tensile force achieved by the VBM^®^ pneumatic pelvic sling with a pressure of 100 mmHg was 73.4 ± 33.1 N. However, at already 45 s after application, the acting tensile forces were reduced to 54.9 ± 28.6 N, after 80 s to 49.4 ± 27.6 N and after 120 s to 46.4 ± 33.1 N. The average peak tensile force achieved at 200 mmHg was 81.5 ± 37.7 N. Here, too, the tensile forces decreased rapidly, as shown in Table 3.

The peak tensile force of 109.9 ± 40.5 N achieved by T-POD^®^ was also reached immediately after application. Here, too, there was a decrease in the acting forces during the defined measurement times, which was more noticeable than in the case of the VBM^®^ pneumatic pelvic sling (Table 3).

The peak tensile force of the cloth sling was on average 105.2 ± 72.6 N. It was noticeable that although there was a rapid reduction in the tensile force after the cloth loop was applied, the other measured values determined over time remained relatively stable.

The SAM Sling^®^ was able to achieve high peak tensile forces, which—analogous to the other pelvic stabilizers tested—became apparent shortly after installation. The rapid decrease in the acting tensile forces was disproportionately strong: After only 45 s, an average of 30.6 ± 25.5 N was recorded. After 80 s, the average values were 29 ± 25.2 N and after 120 s 27.9 ± 24.9 N.

The peak force was reached immediately after application for all pelvic stabilizers. The measured tensile forces decreased rapidly over time for all external, non-invasive pelvic stabilizers. This was most evident with the cloth sling and the SAM Sling^®^. The VBM^®^ pneumatic pelvic sling was able to demonstrate the lowest loss of traction over time at an applied pressure of 200 mmHg.

## 4. Discussion

To our knowledge, this work is the first to describe the biomechanical effects of noninvasive external pelvic stabilizers on the bony structures and intrapelvic volume of pelvic ring injuries. In addition, an epidemiologic analysis of concomitant injuries and autopsy results is performed.

### 4.1. Epidemiology of Pelvic Trauma

The epidemiologic results of this study are consistent with those reported by other authors. The majority of our collective was male (67%). In international study collectives, the polytrauma patient is male in approximately 70% of cases and has an average age of 38 to 47 years [23,24,25]. The determined mean age of 49 years may be due to demographic trends.

In our collective, metropolitan-specific trauma mechanisms emerged with predominantly falls from heights (64%), followed by train rollover trauma (23%) and traffic accidents (13%).

In contrast, data from recent years show that high-altitude falls account for an average of only 20–25% of trauma patient deaths [26,27]. Traffic accidents, to which rollover traumas from trains have often been added, are shown to cause death more frequently, up to 72%, especially in less densely populated areas [27,28].

Parreira et al. illustrated that, for a study population of 103 patients with unstable pelvic injuries, traffic accidents lead with 79% and falls from greater heights with only 17% [29]. These results were confirmed in further studies of patients with unstable pelvic fractures for both the Asian and Australian regions [30,31]. In a previous work, we were able to show already, in a 2010 study collective of trauma-related deaths that falls from heights appear as a frequent death-causing mechanism typical for Berlin [32].

However, our collective included only patients who died in the trauma setting; accordingly, a higher overall injury severity can be assumed. In a large proportion of our patient population (60%, n = 55), death occurred immediately as a result of the accidental event or shortly thereafter (before arrival of the emergency medical services). Consecutively, it can be assumed that the trauma mechanisms identified are causative for the higher overall injury severity in the collective evident in the autopsies.

The majority of patients in the studied collective (87%) died in the prehospital period, which can also be explained by the trauma severity and the high proportion of patients already with certain signs of death showing on finding. In an international comparison, the data on prehospital mortality of trauma patients show a wide range of 41–85% [27,32,33,34,35], which also seems to depend on the localization of the accident. While 72% of patients in rural areas died at the scene of the accident, only 41% did so in urban settings [35].

### 4.2. “Treat First What Kills First” in Pelvic Trauma

The patient collective with unstable pelvic injuries additionally showed very high incidences of injuries to or in the thorax (96%), peripelvic soft tissue injuries (86%), and traumatic brain injury (84%). However, unstable pelvic injury was shown to be the leading source of bleeding in only 19%; thoracic injuries were shown to be the main source of bleeding in our collective (25%).

This represents essential information, which has enormous influence on the prioritization of emergency and surgical management in patients with unstable pelvic injury. Several retrospective studies were found to be congruent with our findings: both Parreira et al. and Poole et al. postulated that although unstable pelvic injury carries a tremendous risk for the development of hemorrhagic shock, the outcome of patients is essentially dependent on concomitant injuries [29,36]. Poole et al. showed that of the 236 patients studied, 18 died, seven because of hemorrhagic shock [36]. However, only one patient was shown to have a pelvic major source of bleeding, whereas the remaining six patients died from extrapelvic major sources of bleeding [36].

For example, in our collective, 12% of patients showed injury to the liver alone as the leading major source of bleeding. Therefore, unstable pelvic injury should always be considered as an indicator of severe internal injury and bleeding until proven otherwise. Consequently, in the case of abdominal major sources of bleeding, for example, due to severe liver injury, clear preference should be given to laparotomy. These findings should be considered in ATLS/ETC concepts and applied to trauma management.

However, it should be kept in mind that in case of a necessary packing of the abdominal cavity in case of surgically uncontrollable bleeding, e.g., from the hepatic stromal area, the surgically stabilized pelvis is a better abutment.

### 4.3. Reproducibility of Genuine Pelvic Trauma with Artificial Pelvic Trauma Models

The case of misinterpretation of a type A fracture of the Ala ossis ileum as unstable pelvic injury shows some limitations of physical examination to determine pelvic ring instability. The definite diagnosis of a type B or type C pelvic ring injury is only possible by radiological imaging [37]. With a sensitivity of up to 93% [38,39], the result of the physical examination should nevertheless be relied upon and, if there is the slightest suspicion of an unstable pelvic injury, the stabilization of the pelvis by an external, noninvasive pelvic stabilizer should already be performed preclinically. The use of a pelvic stabilizer is indicated in cases of mechanically unstable pelvic ring fractures and simultaneous hemodynamic instability. If the pelvis is mechanically stable during the manual examination, pelvic instability is unlikely. If hemodynamics does not stabilize after application, arterial intrapelvic and extrapelvic sources of bleeding must be sought [40].

It is interesting to note that all other patients in our study collective had only Pennal and Tile type C unstable pelvic fractures. Type B fractures were not observed in our collective. The high applied forces during trauma will be causative for this. This fact should be further investigated and, if necessary, lead to a reevaluation of the artificial model and its applicability mostly using artificial type B injuries.

### 4.4. Effect of External Pelvic Stabilization in Real Pelvic Trauma

The results obtained with this study represent the first quantitative data on the effectiveness of external pelvic stabilizers in reducing various parameters of the pelvic area in non-artificial unstable pelvic injuries.

Most of the data published are studies of cadavers with artificially induced pelvic fractures and single case reports [19,20,41,42,43,44,45,46,47]. For the first time, we can present data on quantitative changes in intrapelvic volume, pelvic entrance area, and acting traction forces after the application of external noninvasive pelvic stabilizers to unstable pelvic injuries in real injured patients using computed tomographic imaging and biomechanical measurements.

All external non-invasive pelvic stabilizers achieved a reduction of intrapelvic volume. Therefore, if an unstable pelvic injury is suspected, an external, non-invasive pelvic stabilizer should be applied already in a prehospital setting. The T-POD^®^ succeeded best on average (333 ± 234 cm^3^) but with higher average peak traction force (110 N) compared to the tested devices in this study. The reduction results of the VBM^®^ pneumatic pelvic sling consistently showed significantly better results at an applied pressure of 200 mmHg than at 100 mmHg, with negligible differences in traction force (peak traction force 82 vs. 73 N). In terms of reduction of area in the pelvic entrance plane, the VBM^®^ pneumatic pelvic sling demonstrated the greatest reduction effects in our study at 200 mmHg.

Since the peak traction forces differed only minimally at both pressures, as a result of this study, the recommendation for the adjustment of the recommended pressure level on the manometer of the VBM^®^ pneumatic pelvic sling was adjusted to 200 mmHg by the manufacturer.

The distances between the femoral head and Köhler’s tear figures showed excellent reduction results. In particular, the VBM^®^ pneumatic pelvic sling was able to significantly reduce these parameters.

All pelvic stabilizers succeeded in reducing the symphysis width.

The results regarding the femoral head distance confirmed in each case the results of the area reduction in the pelvic entrance plane. Thus, for clinical practice, femoral head distance can be used as a simple surrogate parameter to control a sufficient reduction of the pelvis by means of pelvic overview imaging in pelvic ring fractures.

The comparison of reduction of the pelvic inlet area and the distances between the femoral heads and the Köhler’s tear figures showed almost congruent results with mostly significantly better results of the pneumatic pelvic sling VBM^®^. Thus, these parameters are shown to be suitable as a measure for assessing the reduction of the anterior pelvic ring.

The relatively small reduction of the dorsal SIJ distance in combination with the predominantly good and congruent reduction results of the parameters of the ventral pelvic ring (femoral head distance, distance between the Köhler’s tear figures, and symphysis width) suggests that external, non-invasive pelvic stabilizers ostensibly influence the ventral pelvic ring.

Assessing the reduction of dorsal SIJ distances for the effectiveness of external noninvasive pelvic stabilizers is severely limited. It can be speculated that especially instabilities in the area of the ventral pelvis can be sufficiently reduced by the application of an external, non-invasive pelvic stabilizer.

Stabilization by means of a conventional sling already has reducing effects. The lack of a pneumatic sling VBM^®^, T-POD^®^, SAM Sling^®^, or other devices designed for this purpose on emergency vehicles or rescue helicopters, should not and must not be considered as an argument against pelvic stabilization already performed prehospital.

Knops et al. demonstrated with their study that the T-POD^®^ required the lowest traction forces compared to the SAM Sling^®^ and the Pelvic Binder for a sufficient reduction of the symphysis width with an average of 43 N [20]. In our study, the T-POD^®^ also required significantly lower peak traction forces than the SAM Sling^®^ (130 N) with an average of 110 N. However, these were still well above the maximum force determined for the VBM^®^ pneumatic pelvic sling (73 and 82 N at 100 and 200 mmHg, respectively).

The characteristic of reaching the highest peak force immediately after application was shared by all four tested external non-invasive pelvic stabilizers. However, our study revealed that the measured tensile forces then decreased rapidly during the time course.

This effect was most evident with the cloth sling and the SAM Sling^®^. For example, the SAM Sling^®^ showed a reduction in tensile forces of almost 80% after just two minutes. Knotting in the ventral pelvis or the nature of the material with consecutive loss of pressure is the main limitation of the use of a cloth sling. The results (high maximum force, rapid loss of force) can be well explained by the narrow design of the cloth sling: The cloth sling shows a width of only a few centimeters in the anterior region—if applied correctly—due to the ventral knotting technique. Interestingly, the high maximum tensile force measured did not have a higher reductive effect on the quality or effectiveness of the reduction of intrapelvic volume or pelvic entrance area. Our study was able to show that the reduction results of the cloth sling were significantly worse than those of the specific pelvic stabilizers. DeAngelis et al. demonstrated a significantly better reduction in symphysis width with the T-POD^®^ compared to the cloth sling [19]. Our results correspond to previously published international literature: Knops et al. showed in their cadaver study that the SAM Sling^®^ required significantly higher traction forces compared to the T-POD^®^ and the Pelvic Binder for sufficient fracture reduction in type B as well as type C pelvic injuries (average 112 N vs. 43 N and 60 N, respectively), whereas the T-POD^®^ succeeded in this reduction already at one third of the traction force [20].

Nevertheless, pelvic stabilization using a cloth sling should not be omitted, as it has proven beneficial effects on fracture reduction and presumably also on patient hemodynamics. If it is possible to use a specific device designed for pelvic stabilization, such as a pneumatic pelvic sling or the T-POD^®^, this should be preferred in any case.

All pelvic stabilizers exhibited the highest peak tensile force shortly after application, with rapid decreases in tensile forces over time (two minutes). This was most evident with SAM Sling^®^ and cloth Sling.

Causes of pressure loss are likely to be pressure distribution in soft tissue, redistribution of interstitial and lymphatic fluid and blood into venous capacity vessels, and reduction of the pelvic injury.

**Figure 7:**
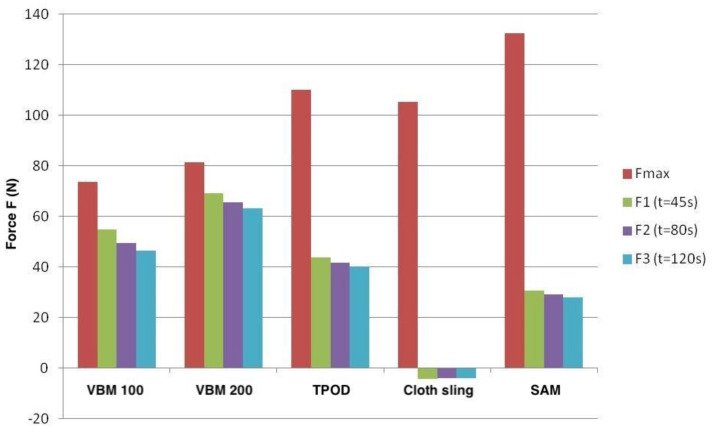
Kinetics of the pelvic stabilizers. Maximum and mean forces achieved at the various predefined times after application of the respective pelvic sling: The SAM Sling^®^ was able to achieve the highest peak tensile forces, but that these fell rapidly and disproportionately. The most constant tensile force of all pelvic slings at the time points examined was demonstrated by the VBM^®^ pneumatic pelvic sling with an applied pressure of 200 mmHg (source: Haussmann [22], modified).

As can be seen from the data in Table 3 and Figure 7, the forces generated by T-POD^®^ and SAM Sling^®^ were only initially the greatest and then quickly decreased. The pneumatic sling VBM^®^, on the other hand, was able to maintain the initially generated forces over a longer period of time. This could be an explanation for the better radiological reduction results.

In terms of intrapelvic volume reduction, the VBM^®^ pneumatic pelvic sling showed a similar volume reduction compared to the T-POD^®^ and predominantly achieved the best results in a comparison of all pelvic stabilizers, despite initially lower force application. Its advantage could be the arrangement of the pneumatic pads, which, when correctly placed, are each placed dorsolaterally on the pelvis. This achieves the compression of both pelvic sides, which could lead to more effective reduction results than purely circumferential application of force to the pelvis.

The T-POD^®^, on the other hand, achieved comparable reduction results, but required a significantly higher maximum force to do so. Over time, however, the reduction in intrapelvic volume is maintained, even under lower pressures.

This could be explained by the vector of the force but also by the amount of the initial applied pressure, which leads to an initial reduction. The lower forces over time could be sufficient to ensure the retention.

Critically, the exact time points of application of the external pelvic stabilizers after fracture or until the CT scan were not part of the data collection. Also, the experimental design could influence the results in that the VBM^®^ pneumatic pelvic sling was applied prior to the T-POD^®^. It remains unclear to what extent some residual retention is maintained on the cadavers due, for example, to rigor mortis.

Bony fractures, possibly also with existing osteoporotic bone structure, were not observed after application of the various devices.

For clinical handling, a readjustment of the pelvic stabilizers may therefore be necessary. The pressure manometer of the VBM^®^ pneumatic pelvic sling has the advantage that the user can measure the pressure and easily readjust.

## 5. Conclusions

Unstable pelvic injuries must be seen predominantly as an indicator of serious, especially thoracic and abdominal, concomitant injuries. In only a fifth of the analyzed cases, the pelvic injury was the leading bleeding source. This has a direct impact on clinical management and prioritization of emergency surgery within multiple trauma management.

Stabilization of unstable pelvic injuries should be performed as soon as possible using specific pelvic stabilizers, such as a T-POD^®^ or pneumatic pelvic sling VBM^®^. A cloth sling should be used only in the absence of specific external pelvic stabilizers. To achieve optimal reduction results using a pneumatic pelvic sling VBM^®^, we advocate an adjustment of the recommended pressure application to 200 mmHg. The extent to which regular readjustment of the pelvic stabilizers is required should be further investigated in future studies.

The specific external, non-invasive pelvic stabilizers pneumatic pelvic sling VBM^®^ and T-POD^®^ could, for the most part, show significantly better reduction results compared to the conventional cloth sling. Therefore, the provision of these specific pelvic stabilizers should be demanded. The DIN standard for the equipment of rescue devices should be adapted.

## Figures and Tables

**Table 1:** Overview of the reduction results based on the mean values and standard deviation in direct comparison of the tested pelvic stabilizers to the native scans (PS1 = pneumatic pelvic sling VBM^®^ with applied pressure of 100 mmHg; PS2 = pneumatic pelvic sling VBM^®^ with applied pressure of 200 mmHg, TP = T-POD^®^; TS = cloth sling, SAM = SAM Sling^®^; SD = standard deviation; * *p* < 0.001; source: Haussmann [22], modified).

	Reduction of Intrapelvic Volume (cm^3^ ± SD)	Reduction Area Pelvic Entrance Level (cm ± SD)	Reduction Symphysis Width (cm ± SD)	Reduction Femoral Head Distance (cm ± SD)	Reduction Distance Köhler’s Tear Figure (cm ± SD)	Reduction SIJ Distance Ventral (cm ± SD)	Reduction SIJ Distance Dorsal (cm ± SD)
PS1	268 * ± 185	34 * ± 29	1.1 ± 1.9	2.5 ± 1.3	2 ± 1.2	1.2 ± 0.9	0.8 ± 2
PS2	306 * ± 176	35 * ± 29	1.2 ± 2	2.9 ± 1.4	2.3 ± 1.2	1.4 ± 1	0.5 ± 1.3
TP	333 * ± 234	28 * ± 24	1.1 ± 1.8	2.1 ± 1.4	1.6 ± 1.1	1.1 ± 0.9	0.36 ± 1
TS	186 * ± 222	18 * ± 18	0.6 ± 1	1.1 ± 1	0.9 ± 0.8	0.7 ± 0.6	0.4 ± 1
SAM	184 ± 114	14 ± 8	0.06 ± 0.1	1.1 ± 0.5	0.9 ± 0.6	0.5 ± 0.4	-

**Table 2:** Overview of the reduction results based on the mean values and standard deviation in direct comparison of the tested pelvic stabilizers to the native scans (PS1 = pneumatic pelvic sling VBM^®^ with applied pressure of 100 mmHg; PS2 = pneumatic pelvic sling VBM^®^ with applied pressure of 200 mmHg, TP = T-POD^®^; TS = cloth sling; SD = standard deviation; source: Haussmann [22], modified).

	PS1	PS2	TP	TS
P1	x			
P2	PV (*p* = 0.002) PA (*p* < 0.001) FH (*p* < 0.001) KT (*p* < 0.001) SIJv (*p* < 0.001)	x		
TP	KT (*p* < 0.05)	PA (*p* = 0.004) FH (*p* = 0.001) KT (*p* = 0.001) SIJv (*p* < 0.05)	x	
TS	PV (*p* = 0.01) PA (*p* < 0.001) SW (*p* < 0.005) FH (*p* < 0.001) KT (*p* < 0.001) SIJv (*p* < 0.001)	PV (*p* < 0.001) PA (*p* < 0.001) FH (*p* < 0.001) KT (*p* < 0.001) SIJv (*p* < 0.001)	PV (*p* < 0.001) PA (*p* < 0.001) FH (*p* < 0.001) KT (*p* < 0.001) SIJv (*p* < 0.001)	x

**Table 3:** Mean values of the acting tensile forces of the five measurements with each of the four pelvic slings (PS1 = pneumatic sling VBM^®^ with pressure of 100 mmHg, PS2 = pneumatic sling VBM^®^ with pressure of 200 mmHg; TP = T-POD^®^; CS = cloth sling; SAM = SAM Sling^®^) at the defined time points (Fmax = maximum force; t1 = 45 sec after application; t2 = 80 sec after application; t3 = 120 s after application; source: Haussmann [22], modified)).

	PS1	PS2	TP	CS	SAM
Fmax [N]	73.4	81.5	109.9	105.2	132.3
t1 [N]	54.9	69	43.8	−4.4	30.6
t2 [N]	49.4	65.5	41.6	−4	29
t3 [N]	46.4	63.2	40.1	−4	27.9

## Limitations

Regarding the results of the SAM Sling^®^ data collection, we explicitly point out the small number of cases used to investigate its properties compared to the other devices. Due to this methodological deficiency, we therefore distance ourselves from a recommendation of a model.
